# Structure–reactivity based control of radical-mediated degradation in thiol–Michael hydrogels†

**DOI:** 10.1039/d5tb01237f

**Published:** 2025-07-25

**Authors:** Bruce E. Kirkpatrick, Miranda T. Rubio, Tvishi Yendamuri, Naomi V. Elmer, Danielle S. W. Benoit, C. Allan Guymon, Kristi S. Anseth, Tayler S. Hebner

**Affiliations:** a Department of Chemical and Biological Engineering, University of Colorado Boulder 596 UCB Boulder CO 80309 USA; b BioFrontiers Institute, University of Colorado Boulder 596 UCB Boulder CO 80309 USA; c Medical Scientist Training Program, University of Colorado Anschutz Medical Campus 13001 East 17th Place Aurora Colorado 80045 USA; d Davidson School of Chemical Engineering, Purdue University 480 Stadium Mall Drive West Lafayette IN 47906 USA thebner@purdue.edu; e Department of Biochemistry, University of Colorado Boulder 596 UCB Boulder CO 80309 USA; f Department of Chemical and Biological Engineering, Brigham Young University Provo UT 84604 USA; g Department of Bioengineering, University of Oregon 6231 University of Oregon Eugene OR 97403 USA

## Abstract

Thiol–Michael addition reactions are widely used for forming cytocompatible and well-defined hydrogels. Numerous types of Michael acceptors have been implemented in these reactions; while maleimides enable rapid crosslinking under physiological conditions and are commonly used for their simplicity, slower-reacting electrophiles such as vinyl sulfones and acrylates offer distinct advantages including improved network homogeneity and ease of handling because of the slower reaction rates. Additionally, thiol–acrylate adducts are hydrolytically labile, whereas thiol–vinyl sulfone adducts are comparably more stable in aqueous environments. Building on our previous work demonstrating radical-mediated degradation of thiol–maleimide hydrogels, we sought to determine whether other thiol–Michael adducts are similarly susceptible to cleavage by radical species. Using both linear and network-forming polymer systems, we found that both Michael-adduct types undergo radical-mediated degradation to varying extents. Furthermore, acrylates are far more prone to radical homopolymerization, enabling semi-orthogonal degradation modes in hydrogels, wherein hydrolytic and radical responses are independently programmed according to the chemical structure and stoichiometric excess of the Michael acceptor. Extending the results of these findings in networks synthesized *via* thiol–Michael addition, we also observed similar radical-mediated degradation behavior in thiol–norbornene networks formed *via* thiol–ene photopolymerization, suggesting that even electron-rich thioethers are degradable under sufficiently aggressive initiation conditions where the concentration of radicals exceeds that of the crosslinks. Together, these results extend the chemical space for engineering hydrogels with variable degradation profiles and illustrate design principles for tuning material responses to multiple chemical stimuli.

## Abstract 2

We report radical-mediated degradation of hydrogels formed by thiol–Michael addition of acrylates and vinyl sulfones and explore the variable susceptibility to free-radical polymerization and hydrolytic stability of these crosslinkers.

## Graphical abstract

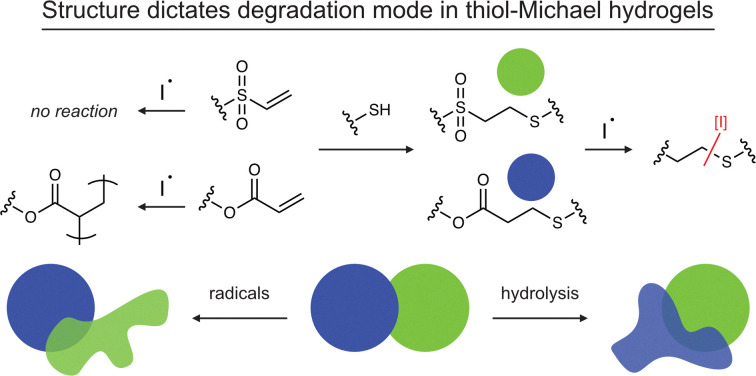

## Introduction

Degradable hydrogels provide powerful platforms for designing materials with user-defined temporal control over polymer network structure. Degradability is often leveraged in soft material systems for a wide range of applications, from templated fabrication and the release of embedded cargo to the removal of sacrificial elements and dynamic modulation of matrix mechanics.^1,2^ Among the strategies for hydrogel formation, thiol–Michael addition reactions are particularly attractive due to their compatibility with aqueous environments and high efficiency with a wide range of commercially available monomers. The versatility of this chemistry has led to its widespread use in the design of polymer networks with tailored mechanical, chemical, and degradation properties.^3^ However, the long-term stability and stimuli-responsiveness of these networks depend on the substituents of the Michael adduct and the surrounding chemical environment.^4^

Maleimides, acrylates, and vinyl sulfones are all electrophilic alkenes commonly used to form thiol–Michael hydrogels, each offering distinct advantages with respect to reactivity, stability, and practical handling. Maleimides react rapidly with thiols under physiological conditions, enabling fast gelation but often leading to network heterogeneity because of the limited working time for mixing.^5–7^ In addition, the diverse reactivity of maleimides can lead to a variety of side reactions depending on environmental conditions.^8^ In contrast, acrylates and vinyl sulfones react more slowly and selectively with thiols, which can improve homogeneity and user control over the timing of network formation. However, dissimilarities in alkene substitution can also influence long-term adduct stability: thiol–maleimide adducts are susceptible to retro-Michael and thiol exchange reactions,^9^ while thiol–vinyl sulfone adducts are comparatively more stable in aqueous environments and thiol–acrylate adducts are most prone to hydrolytic cleavage.^10^ Harnessing these differences, previous work utilized rational combinations of acrylate and vinyl sulfone crosslinkers to form thiol–Michael hydrogels with highly predictable degradation profiles for dynamic cell culture applications.^11,12^ While the formation kinetics, relative selectivity, and hydrolytic stabilities of these adducts have been well-studied,^13^ less is known about how structural differences between Michael acceptors influence their susceptibility to radical cleavage, a stimulus class that offers a temporally controlled mechanism and complements other conventional degradation cues like hydrolysis. Recently, we demonstrated that thiol–maleimide adducts undergo cleavage in the presence of photoinitiated and redox-generated radicals, revealing a surprising new method for degrading these widely used hydrogel systems in an on-demand manner.^14^ Motivated by these findings, we sought to investigate degradation behavior of other thiol–Michael adducts - specifically, those formed from acrylates and vinyl sulfones.

Herein, we investigate the susceptibility of thiol–acrylate and thiol–vinyl sulfone hydrogels to radical-mediated degradation. Using linear polymers linked by Michael adducts, we identify chemical signatures consistent with thioether bond cleavage and the formation of new adducts incorporating radical initiator fragments. Rheological analysis of hydrogels crosslinked by Michael addition reveal that vinyl sulfone-based networks degrade more efficiently than their acrylate-based counterparts when exposed to photoinitiated radicals and exhibit more pronounced concentration-dependent responses to radical dose in bulk systems. Leveraging these material chemistries, we next show spatiotemporal control over gel degradation, and then exploit differences in the hydrolytic stability between acrylates and vinyl sulfones and their capacity for homopolymerization to selectively stabilize or erode gels with orthogonal stimuli (*i.e.*, redox-initiated radicals and sodium hydroxide). Extending our findings from the Michael addition-based systems, we further show that this radical-mediated cleavage behavior applies to thioether bonds formed *via* radical thiol–ene addition. Collectively, these experiments demonstrate an orthogonal and pathway-independent strategy for programming material degradation and cargo release in synthetically simple and commonly used hydrogel formulations. Our results highlight important structure–reactivity considerations for polymer networks subjected to radical stimuli.

## Results and discussion

**Fig. 1:**
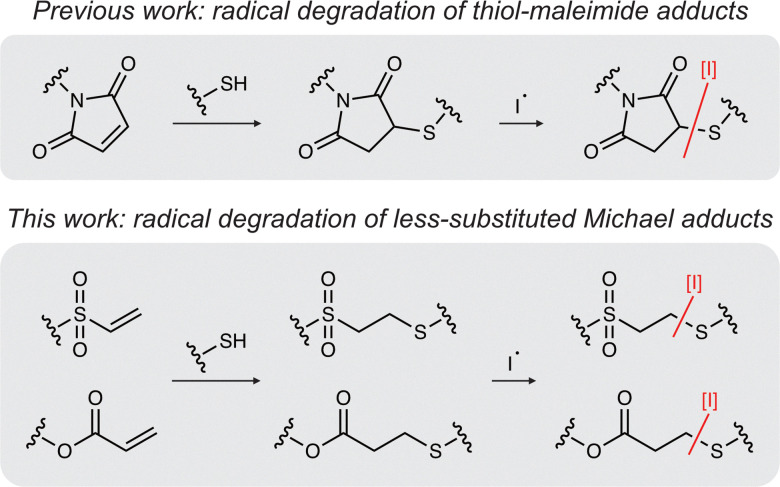
Thiol–Michael adducts are susceptible to radical-mediated degradation. Previously, thiosuccinimides formed by thiol-maleimide addition were shown to be cleaved by radical initiator species (I*). In this report, similar radical-mediated degradations are studied in the context of Michael adducts formed by vinyl sulfones and acrylates.

There are important structural and electronic distinctions between various electrophilic alkenes commonly used in thiol–Michael reactions. Maleimides contain a cyclic imide flanking the Michael acceptor, whereas acrylates and vinyl sulfones present terminal alkenes adjacent to electron-withdrawing ester or sulfone groups, respectively. These differences influence not only reaction kinetics and hydrolytic stability but also the chemical reactivity of the resulting thioethers and, therefore, their susceptibility to radical-mediated bond cleavage. Specifically, thiol–maleimide adducts contain a thiosuccinimide that may be especially susceptible to radical-mediated cleavage due to the formation of a relatively stable secondary carbon-centered radical upon C–S bond homolysis. In contrast, cleavage of thioethers formed from acrylates or vinyl sulfones generate less stable primary carbon-centered radicals, potentially reducing their susceptibility to degradation. Additionally, as acrylates and vinyl sulfones are distinguished by their electron-withdrawing groups, the associated electronic effects are likely to influence their comparative reactivity toward radicals. In our previous report,^14^ we showed that thiosuccinimide crosslinks formed by Michael addition are readily cleaved by radicals, prompting the exploration of this reaction with acrylate and vinyl sulfone-derived thiol–Michael adducts (Fig. 1). Using a combination of linear polymers and crosslinked networks encompassing these less-substituted Michael adducts, we performed degradation studies to quantify the extent of radical-mediated thioether cleavage across different radical sources and material contexts.

Linear polymers were used to identify radical-mediated degradation products using well-defined poly(ethylene glycol) (PEG) monomers reacted in aqueous solutions. Monofunctional 1 kDa PEG macromers functionalized with either acrylate or vinyl sulfone were reacted with 2 kDa PEG–dithiol at a 1 : 1 molar ratio of Michael acceptor to thiol and a final adduct concentration of 10 mM in deionized water. Reactions were catalyzed using 0.05 or 0.3 M triethanolamine and carried out in the presence of 2 wt% lithium phenyl-2,4,6-trimethylbenzoylphosphinate (LAP) as a source of photoinitiated radicals. All monomer, initiator, and catalyst components were mixed together to facilitate a one-pot reaction sequence in which the Michael addition reaction was carried out in the dark and subsequent light exposure generated radicals to degrade the Michael addition products. Following a 30-minute reaction period at room temperature to allow the Michael addition reaction to reach completion, disappearance of characteristic alkene and thiol peaks in ^1^H NMR confirmed Michael adduct formation (Fig. S1, ESI†). Samples were then irradiated with 365 nm light at 20 mW cm^−2^ for 20 minutes to generate radicals and induce degradation.

**Fig. 2:**
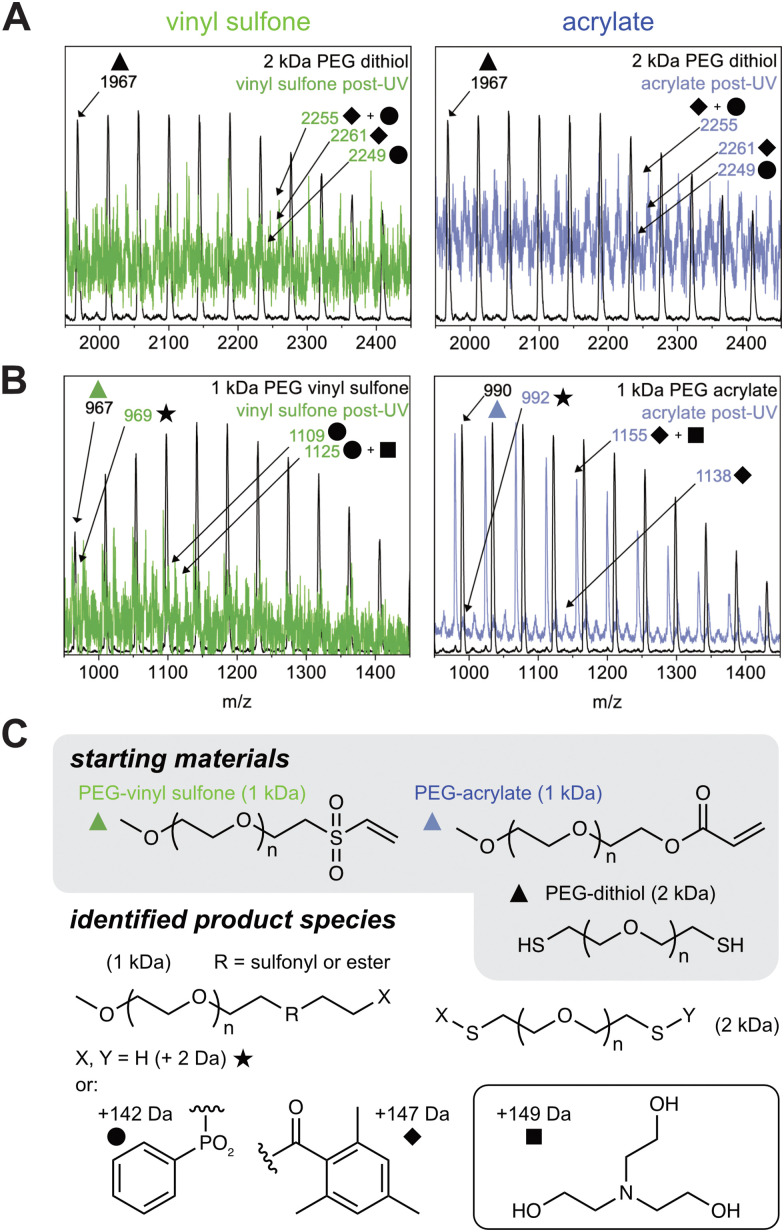
Analysis of radical-mediated degradation products in linear thiol–Michael polymers. (A) MALDI-TOF spectra highlighting 2 kDa species (*i.e.*, PEG-dithiol), including starting material (black) and degradation products (green for vinyl sulfone, blue for acrylate) formed *via* recombination with LAP-derived radical fragments. (B) Spectra showing 1 kDa species (*i.e.*, mPEG-alkene), including starting materials (black) and products (green for vinyl sulfone, blue for acrylate) consisting of hydrogen-terminated species and products formed by recombination with initiator fragments. (C) Representative chemical structures corresponding to major degradation products assigned in panels A and B.

To identify degradation products, MALDI-TOF mass spectrometry was performed before and after photoinitiated radical generation. Prior to UV exposure, both acrylate and vinyl sulfone reactions produced the expected bifunctional Michael adducts at ∼4 kDa, with additional signals corresponding to unreacted 1 kDa and 2 kDa starting materials. After irradiation, the higher molecular weight species appeared to be partially depleted, and new peaks emerged in the 1–3 kDa range (Fig. S2, ESI†). In both systems, we observed products with molecular weights corresponding to PEG–dithiol capped with LAP-derived fragments, suggesting homolytic C–S bond cleavage followed by radical recombination (Fig. 2(A)). A second common degradation product matched the 1 kDa PEG starting material with two added hydrogen atoms, consistent with hydrogen abstraction by the Michael acceptor as a possible radical-terminating pathway (Fig. 2(B)). Interestingly, we observed peaks in the acrylate system corresponding to new adducts with the benzoyl-centered LAP fragments, while vinyl sulfones appeared to favor recombination with the phosphinate-centered fragments (assignments shown in Fig. 2(C)). Both the acrylate and vinyl sulfone-based thioethers resulted in only partial degradation under these conditions, leaving some products in the range of ∼4 kDa. However, thiol–maleimide bonds appeared to undergo complete scission based on the disappearance of these peaks (Fig. S4, ESI†). This result is consistent with the formation of a secondary carbon radical that is significantly more stable (*i.e.*, more readily generated) than either of the primary radicals formed from acrylate or vinyl sulfone adducts. These differences in degradation behavior between the three Michael addition systems may arise from the relative stabilities of the transient radical intermediates.

Of note, the inclusion of triethanolamine to facilitate the Michael addition reaction also impacted the degradation profiles. MALDI spectra revealed degradation products consistent with association between triethanolamine and PEG-based fragments. These included masses corresponding to the 1 kDa starting material combined with triethanolamine, as well as adducts containing both triethanolamine and LAP-derived fragments. We hypothesize that triethanolamine may participate in radical quenching reactions and/or form non-covalent adducts during MALDI. Such behavior is commonly observed with ionizable species, highlighting the complexity of interpreting degradation products in radical-rich environments where other side reactions may be taking place. Notably, these peaks were less pronounced compared to non-complexed peaks when the triethanolamine concentration was reduced to 0.05 M (Fig. S3, ESI†). Taken together, these linear polymer studies suggest that while thiol–acrylate and thiol–vinyl sulfone adducts are less susceptible to radical-mediated cleavage than thiol–maleimide adducts, both undergo bond scission under sufficiently a high radical generation rate. The chemical nature of the alkene and the identity of the initiator fragments likely influence both the efficiency and the pathways of degradation, implying that different Michael acceptors might be used to tailor the stability and responsiveness of network-forming materials.

**Fig. 3:**
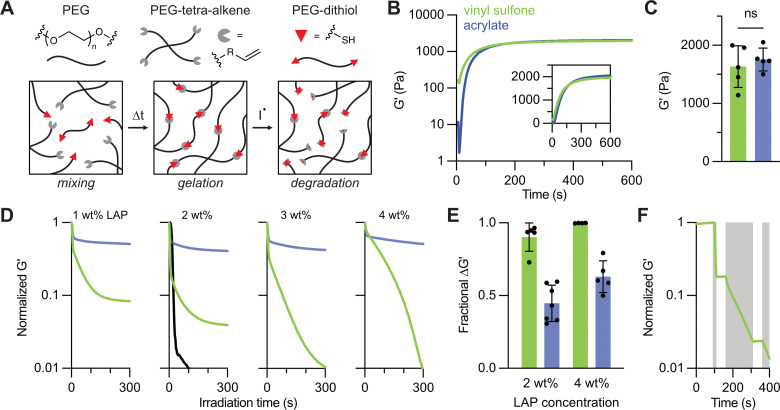
Radical-mediated degradation of thiol–Michael hydrogels formed by vinyl sulfones and acrylates. (A) Schematic of network formation *via* thiol–Michael addition and subsequent degradation by radical species (I*). (B) *In situ* rheology during gelation shows similar crosslinking kinetics for vinyl sulfone (green) and acrylate (blue) hydrogels with (C) comparable plateau storage moduli. (D) *In situ* photodegradation profiles at varying LAP concentrations (1–4 wt%) reveal dramatically increased radical susceptibility in vinyl sulfone gels. For the 2 wt% condition, a matched thiol–maleimide gel (black) is included. The change in degradation profile between 3 and 4 wt% LAP for the vinyl sulfone hydrogels may relate to the optical thickness of these samples. (E) A summary of degradation extent at 2 and 4 wt% LAP demonstrates the increased resistance of thiol–acrylate gels to radical-mediated softening. (F) Pulsed light exposure of a vinyl sulfone gel (4 wt% LAP) confirms that degradation proceeds only during illumination (gray time regions), illustrating temporal control.

To evaluate radical-mediated degradation in crosslinked materials, hydrogels were formed by reacting 4-arm 20 kDa PEG macromers functionalized with acrylate or vinyl sulfone endgroups with 2 kDa PEG–dithiol in a 6 wt% polymer solution (10 mM Michael adducts) and a 1 : 1 stoichiometric ratio of thiol to alkene. Hydrogel formulations also contained 0.3 M triethanolamine and varying concentrations of LAP (1–4 wt%). Network formation was monitored by *in situ* oscillatory shear rheology before and during irradiation with 365 nm light at 10 mW cm^−2^. The gels were first allowed to crosslink in the dark *via* thiol–Michael addition for 10 minutes, then exposed to light to initiate radical-mediated degradation (Fig. 3(A)). Both acrylate- and vinyl sulfone-based hydrogels formed robust networks within seconds after combining the thiol and alkene macromers, exhibiting similar network formation profiles (Fig. 3(B)). After 10 minutes, both systems reached comparable plateau storage moduli (*G*′) of 1630 ± 360 Pa for vinyl sulfone gels and 1760 ± 200 Pa for acrylate gels (Fig. 3(C)), consistent with the similar initial macromer functionality and molecular weight.

Upon UV exposure, the storage modulus decreased in all networks (Fig. 3(D)). At all tested LAP concentrations, acrylate gels showed moderate degradation (30–60% reduction in storage modulus), while vinyl sulfone gels softened substantially more than acrylate gels at matched concentrations of initiator. For example, at 1 wt% LAP, vinyl sulfone gels partially degraded, exhibiting >80% reduction in storage modulus, while acrylate gels reached ∼30% reduction in modulus. At 2 wt%, vinyl sulfone gels reached >90% reduction in modulus, and acrylate gels approached 50% softening. When 3 wt% LAP was included, the vinyl sulfone materials reached the reverse gel point while the acrylate gels did not degrade further. Increasing the initiator concentration to 4 wt% LAP resulted in consistent reverse gelation of vinyl sulfone gels, but the acrylate gels only exhibited marginal increases in degradation over other LAP concentrations (Fig. 3(E)). These results emphasize the importance of alkene structure and its influence on degradation efficiency in response to high radical doses. This network degradation behavior suggests that differences between the ester moiety of the acrylate and the sulfonyl of the vinyl sulfone make the vinyl sulfone more susceptible to carbon radical formation and bond cleavage, which could relate to electron-withdrawing effects.^15,16^ However, since some reports suggest similar Hammett values (a metric for assessing the electron-withdrawing or donating effects of a substituent) between the ester and sulfonyl functionalities of the acrylate and vinyl sulfone, respectively, these findings indicate that local radical stability and reactivity may be influenced by factors beyond simple inductive effects.^17^ Relating these findings to previous results, maleimide-based gels (included as a reference at 2 wt% LAP, with lower concentrations characterized in prior studies^14^) expectedly showed rapid and complete degradation upon light exposure, demonstrating greater susceptibility to radical-mediated bond cleavage than the other two alkenes.

Expanding on these observations, measurement of photoinduced creep in acrylate and vinyl sulfone networks containing 4 wt% LAP further revealed that vinyl sulfone gels exhibit greater time-dependent strain during irradiation (Fig. S5, ESI†), corroborating the previous photodegradation studies focused on tracking modulus. Considering the distinct differences in the susceptibility of these crosslinking chemistries to radical cleavage, we also verified that combining equal proportions of each alkene-bearing macromer in a thiol–Michael gel resulted in an intermediate degradation profile (Fig. S6, ESI†), further endorsing the proposed structure–reactivity relationship wherein each component additively contributes to the overall extent of degradation. Importantly, these studies suggest the ability for user-controlled temporal degradation of hydrogels by simple modulation of light. To demonstrate this, the light was toggled on and off during irradiation of a vinyl sulfone gel containing 4 wt% LAP while following changes in the material properties. Under these conditions, the storage modulus only decreased during illuminated intervals with no appreciable change in the dark (Fig. 3(F)), supporting that degradation proceeds only in the presence of photoinitiated radicals.

To assess potentially confounding effects from light intensity, optical density, and the presence of unreacted functional groups, several additional rheological analyses were performed. First, while the rate of degradation changed in correspondence with 10-fold higher or lower light intensities, the final extent of softening remained unchanged (Fig. S7, ESI†), suggesting that radical-mediated C–S bond homolysis is not strongly dependent on the rate of radical production (as controlled by light intensity) under these conditions. High LAP concentrations (3–4 wt%, corresponding to 102–136 mM) led to ∼40% attenuation in light at a depth of 200 μm (Fig. S8, ESI†), but this did not preclude network degradation in 200 μm-thick hydrogels when sufficient radicals were generated. Further, hydrogels with half the thickness of these samples showed similar degradation profiles (Fig. S9, ESI†). Additionally, to determine whether degradation could be tuned by the presence of unreacted functional groups, off-stoichiometry gels were prepared with either excess thiol (2 : 1 thiol : alkene) or excess alkene (1 : 2). In the presence of 4 wt% LAP, excess thiol did not substantially increase degradation (Fig. S10A, ESI†), suggesting that thiyl radicals do not play a dominant role in promoting cleavage or exchange of thiol–Michael adducts, distinct to prior work of other thioether-based polymer networks and disulfide and allyl sulfide linkages.^18–24^ In contrast, gels with excess alkene showed different behavior. Acrylate-based systems stiffened following irradiation, while vinyl sulfone gels exhibited their typical degradation profile (Fig. S10B, ESI†). This result is consistent with the known tendency of acrylates, but not vinyl sulfones, to undergo radical homopolymerization under these conditions. To validate this assumption, we irradiated acrylate and vinyl sulfone macromers under the same conditions as before (10 mM alkene, 4 wt% LAP) but without thiol to eliminate the possibility of Michael addition; only the acrylate samples showed gelation upon light exposure, confirming selective homopolymerization compared to vinyl sulfone gels (Fig. S11, ESI†).

**Fig. 4:**
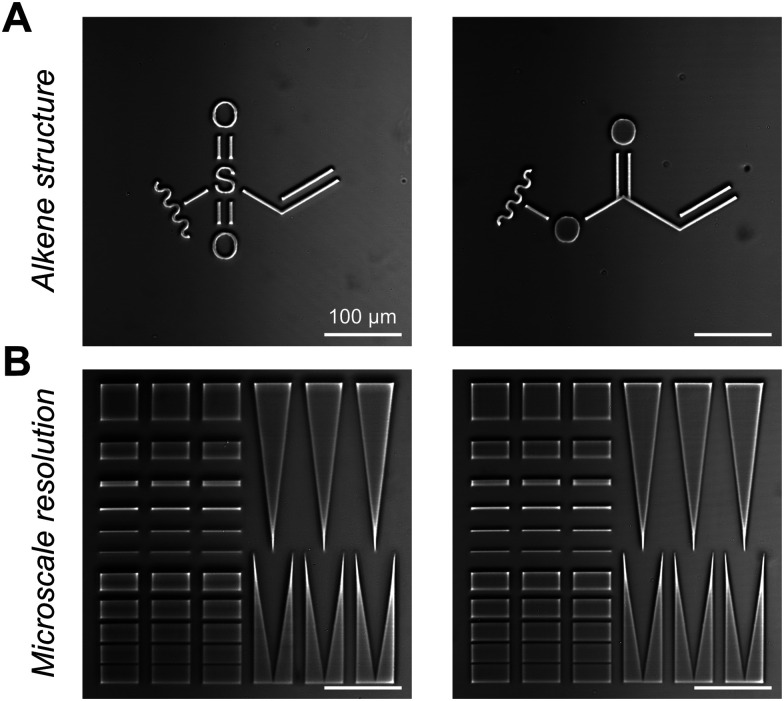
Microscale patterning of thiol–Michael hydrogels. (A) Photopatterns of alkene structure formed by rastered laser exposure in vinyl sulfone (left) and acrylate (right) gels containing 4 wt% LAP. (B) Resolution test demonstrating high-fidelity formation of both positive and negative features at the micron scale for both systems (vinyl sulfone left, acrylate right). Quantification shown in Fig. S12 (ESI†).

We evaluated if the differences in radical susceptibility suggested by photorheology would influence the fidelity of spatial patterning by rastering a laser across the acrylate and vinyl sulfone hydrogels using a confocal microscope. Both materials were modified with sharply defined features with minimal degradation in the unpatterned regions (Fig. 4(A)). The pattern precision was comparable between the two gel types, as determined in a resolution test (Fig. 4(B) and Fig. S12, ESI†). Although bulk rheological degradation differed significantly between formulations, both supported consistent and high-quality microscale patterning, with *R*^2^ ≥ 0.99 for both positive and negative features from 1–50 μm, indicating a strong correlation between the intended and actual pattern sizes. This unexpected similarity in patterning fidelity, despite pronounced differences in bulk degradation, suggests that local, micron-scale effects may play an important role during photodegradation.

**Fig. 5:**
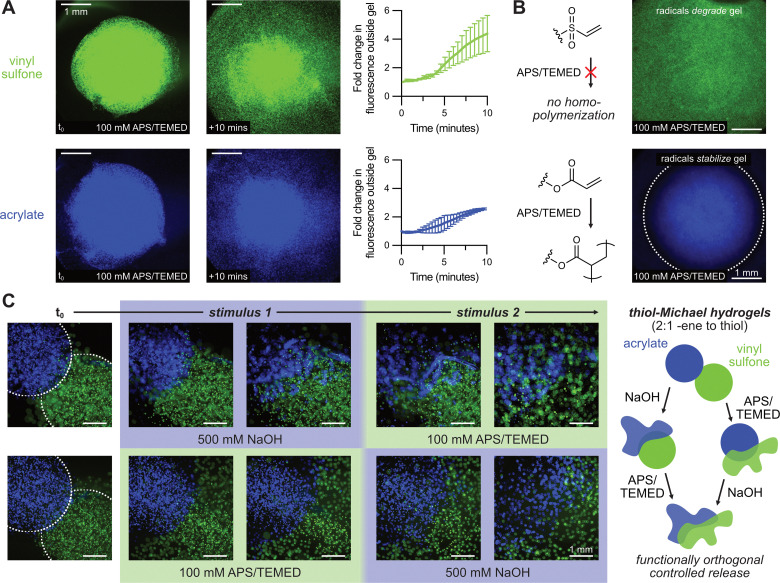
Orthogonal degradation of thiol–Michael hydrogels by redox-initiated radicals and hydrolysis. (A) Fluorescent microbeads embedded in thiol–Michael hydrogels are rapidly released upon introduction of redox initiator (APS/TEMED), confirming radical-mediated degradation of both vinyl sulfone (green) and acrylate (blue) gels. (B) In gels formed with excess alkene (2 : 1 alkene : thiol), redox-initiated radicals selectively degrade vinyl sulfone gels while acrylate gels remain intact 40 minutes after the addition of APS/TEMED, consistent with acrylate homopolymerization-based network stabilization. (C) Sequential treatment with radicals or base revealed functionally orthogonal degradation responses in 2 : 1 alkene : thiol gels, wherein NaOH selectively degrades acrylate gels while preserving vinyl sulfone networks, and APS/TEMED causes the inverse, enabling pathway-independent and chemically encoded degradation modes.

We next explored whether the degradation behaviors observed with photoinitiated radicals extended to redox-initiated systems, using ammonium persulfate (APS) in the presence of *N*,*N*,*N*′,*N*′-tetramethylethylenediamine (TEMED) as initiator, as we previously found that maleimide-crosslinked gels also degraded under these conditions.^14^ To monitor degradation, fluorescent beads were encapsulated in both acrylate and vinyl sulfone gels, which were released upon APS addition over the time course of the 10 minutes (Fig. 5(A) and Movie S1, ESI†), consistent with radical-mediated degradation. Motivated by the rheological results showing acrylate stiffening under excess alkene conditions, we hypothesized that acrylate and vinyl sulfone gels could be selectively stabilized or degraded using redox radicals. Using off-stoichiometry networks (2 : 1 alkene : thiol), we found that radical exposure selectively degraded vinyl sulfone gels while leaving acrylate gels structurally intact (Fig. 5(B) and Movie S2, ESI†). This divergent response is consistent with acrylate radical homopolymerization, which preferentially stabilizes these gels under radical-rich conditions while vinyl sulfones, which lack efficient homopolymerization pathways, remain vulnerable to C–S bond cleavage. These findings underscore the potential for exploiting inherent chemical differences between Michael acceptors to tune network architecture under identical radical stimuli.

To expand upon this principle of selective reactivity, we demonstrated radical-mediated degradation and hydrolytic cleavage in a sequence-independent and functionally orthogonal manner. Given the known relative hydrolytic lability of acrylates compared to vinyl sulfones, we posited that selective degradation of either formulation could be achieved by first applying NaOH or APS/TEMED to gels containing excess alkene. NaOH treatment resulted in selective erosion of the acrylate gel, while the neighboring vinyl sulfone gel remained intact until its subsequent degradation with the introduction of APS/TEMED. Conversely, initial radical exposure *via* APS/TEMED degraded the vinyl sulfone gel while preserving the structural integrity of an adjacent acrylate gel (through acrylate homopolymerization) before NaOH-mediated hydrolysis (Fig. 5(C)). These experiments demonstrate a simple strategy for encoding dual-responsiveness into hydrogel formulations based on orthogonal degradation pathways and chemical structure alone, without requiring synthetically challenging functional groups or spatial patterning steps.

As a final observation, we found that even thiol–norbornene gels, often regarded as stable in the presence of radicals, exhibited signs of radical-mediated softening at high initiator concentrations. Using photorheology, we observed photoinitiated radical-controlled decreases in modulus following photopolymerization with high LAP concentrations, and APS/TEMED treatment triggered fluorescent bead release from these networks (Fig. S13, ESI†). While these results are the subject of rigorous future study at much lower radical concentrations, these preliminary findings suggest that some systems lacking thiol–Michael linkages but containing other C–S bonds may undergo radical-mediated cleavage reactions, underscoring the broader relevance of radical dose and alkene identity in dictating polymer network fate.

Although the hydrogel degradation strategies presented here rely on stimuli that are not cytocompatible (*i.e.*, high concentrations of radicals and base), they nonetheless open the door to a wide range of useful applications. For instance, in soft matter and material templating contexts where photodegradability is desired prior to cell seeding, thiol–Michael adducts offer a synthetically simple, modular, and commercially accessible route to PEG hydrogels with spatiotemporal degradation responses. This contrasts with other systems that introduce photolability to PEG-based materials *via* multi-step chemical reactions and purification steps, such as *ortho*-nitrobenzyl,^25^ coumarin,^26^ bimane,^27^ or triazolinedione chemistries.^28^ Additionally, thiol–Michael and radical reactions can be wavelength-gated using lambda-orthogonal photobase and photoinitiator systems,^29,30^ potentially allowing for combined light-based additive and subtractive manufacturing in a single resin.

Beyond photoresponsive biomaterials, there is also interest in designing hydrogels with well-controlled hydrolytic responses *via* the incorporation of specific linkers, including aromatic esters, carbamates, carbonates, beta-amino esters, and acetals.^31–35^ As such, rational combinations of maleimide, acrylate, and vinyl sulfone functionalities offer an alternative and tunable platform for controlling the kinetics and modes of degradation by both hydrolysis and radical-mediated cleavage. The proposed framework of material chemistries could support several emerging directions in hydrogel engineering, including the creation of materials that encode mechanical or topographic information compatible with sequential or orthogonal erasure,^36,37^ the development of flowable and degradable valve-like materials in fluidic systems,^38,39^ and the design of sacrificial hydrogel elements for templating structures *via* techniques such as viscous fingering or chaotic mixing.^40–42^ The ability to program gels with independently addressable degradation pathways through simple, well-characterized monomers that are widely employed as biomaterials creates a powerful toolkit for building new types of responsive soft materials.

Despite the versatility of the presented degradable thiol–Michael systems, important questions remain, including deeper study of the underlying reaction mechanisms. In both photoinitiated and redox-initiated systems, cleavage was mediated by radicals derived from LAP or APS/TEMED, respectively. The distinct structures and reactivity of these radical species likely contribute to the degradation efficiency and selectivity observed across different thiol–Michael adducts. As an example, we were unable to observe complete degradation in the on-stoichiometry acrylate system even with 4 wt% LAP, but this network underwent reverse gelation at a similar concentration of APS/TEMED. Unfortunately, there are a limited number of initiator systems that are soluble at such high concentrations in water, but other solvents and material systems will allow for future studies of alternative initiators and more rigorously probe the role of different initiator species (*i.e.*, carbon- *versus* heteroatom-centered radicals) on degradation mechanisms.^23^

Beyond the Michael adducts studied here, an array of alkenes are employed in materials design as Michael acceptors, including acrylamides,^43,44^ internal and external methacrylates,^45–47^ ether acrylates,^48^ cyanoacrylates,^49^ chalcones,^50^ and benzylcyanoacetamides,^51^ prompting future investigations of whether similar radical susceptibilities exist across this broader chemical space, although some of these alkenes have only limited reactivity as Michael acceptors and may require alternative processing conditions. Likewise, although norbornene-based systems are typically used in the context of hydrolytic or enzymatic degradation,^52^ our observations suggest that under radical-rich conditions, even these electron-rich thioethers may undergo homolysis. These findings suggest avenues for further exploration into the stability of other adducts formed by radical or cationic polymerizations, including bonds between thiols and vinyl esters, allyl and vinyl groups, or alkynes.^53–55^ In this work, our focus was centered on the identity of the alkene, but the structure of the thiol also influences selectivity towards specific alkenes,^56^ as well as adduct stability and degradation behavior.^57–59^ Exploring how thiol electronics and sterics contribute to radical lability may offer further opportunities to tune degradation pathways. Moreover, integration with additional orthogonal degradation mechanisms, such as enzymatic or chemically selective cleavage,^60–62^ would allow for hierarchical gating of network disassembly and more sophisticated materials-based logic,^63^ broadening the utility of these systems for advanced soft matter and biomedical applications.

## Conclusions

This work demonstrates that thiol–Michael adducts formed from acrylates and vinyl sulfones undergo radical-mediated degradation, similar to previously studied thiol-maleimide crosslinks. Using PEG hydrogels as a model system, differences in susceptibility to degradation were uncovered that reflect the electronic and structural features of the Michael acceptor. Other thioethers also exhibited similar radical-mediated degradation behavior, motivating future study into radical lability of a variety of systems. These findings establish a simple and modular approach to programming the degradation of thiol–Michael hydrogels with spatiotemporal control, orthogonal stimuli, and tunable responsiveness based on alkene structure and formulation stoichiometry. More broadly, this study offers a versatile framework for engineering responsive soft materials using easily accessible chemistries and introduces new opportunities for designing degradable hydrogels.

## Methods

### Materials

No new materials were synthesized for this work. PEG dithiol, mPEG–alkenes, multi-arm PEG vinyl sulfone, acrylate, and maleimide were purchased from JenKem Technology USA. PEG–norbornene was prepared as previously described.^20^ Fluorescent microbeads (FluoSpheres) were purchased from ThermoFisher Scientific. All other reagents were purchased from Sigma-Aldrich and used as received.

### Linear polymer synthesis

Linear polymers were synthesized using 2 kDa PEG dithiol in combination with monofunctional 1 kDa PEG macromers functionalized with acrylate, vinyl sulfone, or maleimide at a thiol to alkene molar ratio of 1 : 1. Macromers were combined in solution (deionized water for vinyl sulfone and acrylate, phosphate buffered saline for maleimide) at a 10 mM functional group concentration. Lithium phenyl-2,4,6-trimethylbenzoylphosphinate (LAP) was added at a concentration of 2 wt% and triethanolamine was added at a concentration of either 0.05 M or 0.3 M. After combining in solution and vortex mixing, the Michael addition reaction was allowed to proceed for 20 minutes at room temperature. To facilitate photodegradation, the solution was then exposed to UV light for 10 minutes (365 nm, 10 mW cm^−2^). Samples were flash-frozen in liquid nitrogen and lyophilized overnight. Dried solids were then used to prepare appropriate solutions for the characterization experiments described below.

### Linear polymer characterization

Matrix-assisted laser desorption/ionization-time of flight (MALDI-TOF) mass spectrometry experiments were performed in linear mode at 90% laser power with a 337 nm, 20 Hz Nitrogen laser (Voyager-DE, Applied Biosystems) using a dithranol matrix (5 mg mL^−1^) and a sodium trifluoroacetate electrolyte (NaTFA, 1 mg mL^−1^). NaTFA solution (1 mg mL^−1^) was mixed in equal parts with linear polymer solutions (5 mg mL^−1^). Acetonitrile was used as a solvent for the dithranol, and water was used for the NaTFA and polymer solutions. Matrix solution was deposited in 0.5 μL aliquots on the sample plate wells and allowed to dry before 1 μL of electrolyte/sample solution was deposited on top of the matrix and dried in air. ^1^H Nuclear Magnetic Resonance (NMR) experiments were performed on a Bruker Avance-500 MHz NMR Spectrometer. Dry linear polymer samples were dissolved in D_2_O at a concentration of 10 mg mL^−1^.

### Hydrogel formation

Hydrogel networks were made from stock solutions of 4-arm 20 kDa PEG–acrylate or vinyl sulfone (20 wt%) and 2 kDa PEG–dithiol (4 wt%) dissolved in 0.3 M triethanolamine in deionized water and various wt% LAP, as indicated throughout the text. These stocks were each diluted 1 : 4 in additional triethanolamine/LAP solution to produce hydrogels at 6 wt% overall solids and 10 mM thiol–Michael crosslinks. After mixing the thiol and alkene macromers, hydrogel-forming solutions had an approximately 30 second handling time before gelation made pipetting impossible. Off-stoichiometry gels were prepared by increasing the excess component while maintaining the 10 mM concentration of thiol–Michael crosslinks at complete conversion of the limiting reagent (*i.e.*, off-stoichiometry gels were >6 wt% total solids). Thiol–maleimide and thiol–norbornene hydrogel precursors were dissolved in phosphate-buffered saline without triethanolamine. For hydrogels prepared for bead release assays, no LAP was included in the formulation and fluorescent beads (1 or 10 μm) were included at a dilution factor of 1 : 1000 for 1 μm beads and 1 : 200 for 10 μm beads. The formation of 200 μm-thick hydrogels was monitored by *in situ* oscillatory shear rheology at 1% strain and 1 rad s^−1^ on a DHR-3 rheometer equipped with a quartz photo-curing plate and an 8 mm parallel plate geometry (TA Instruments). To prevent sample drying, hydrogels were encircled by a ring of mineral oil after the gel point was reached.

### Analysis of hydrogel degradation

Hydrogel degradation was monitored by *in situ* oscillatory shear rheology using the same setup described for hydrogel formation. UV light (365 nm) was supplied using an OmniCure lamp at an intensity of 10 mW cm^−2^ unless otherwise specified. Microscale patterning was performed on a Zeiss LSM 710 confocal microscope as previously described.^64^ Briefly, gels were formed between a glass slide and coverslip, allowed to polymerize for 10 minutes, and then exposed to digital masks encoded as custom.ovl files. Patterning was performed using a 405 nm laser at 100% power (1.6 mW), with a pixel size of 0.42 μm and a dwell time of 25.21 μs. Pattern fidelity was quantified using ImageJ. Hydrogels containing fluorescent microspheres were prepared directly in glass-bottom imaging dishes. Following gelation, 0.2 M TEMED was added to the surrounding medium. Time-lapse fluorescence imaging was performed on a Nikon Ti-2 E inverted microscope, with images acquired every 15 seconds. For isolated bead release studies, 1 μm microspheres were used, while 10 μm beads were used for assays comparing multiple gels within the same imaging field. Redox-initiated degradation was induced by adding an equal volume of 0.2 M APS to yield a final concentration of 0.1 M APS/TEMED. In orthogonal release assays, gels designated for hydrolytic degradation were first equilibrated in deionized water and then treated with an equal volume of 1 M NaOH (final concentration 0.5 M). Bead release was defined as the downward transit of fluorescent microspheres out of the imaging plane, indicating dissolution of the encapsulating hydrogel that had suspended the beads above the dish surface.

## Conflicts of interest

There are no conflicts of interest to declare.

## Supplementary Material

TB-013-D5TB01237F-s001

TB-013-D5TB01237F-s002

TB-013-D5TB01237F-s003

## Data Availability

All data needed to evaluate the conclusions in the paper are present in the paper and/or the ESI.† Raw data are available from the authors upon reasonable request.
