# Supplementary material for: Structure–reactivity based control of radical-mediated degradation in thiol–Michael hydrogels
Source: J Mater Chem B. 2025 Jul 25;13(33):10177–86. doi: 10.1039/d5tb01237f (PMC12323595; doi:10.1039/d5tb01237f)
Supplement: TB-013-D5TB01237F-s003 [file TB-013-D5TB01237F-s003.pdf]

Supplementary Information (SI) for Journal of Materials Chemistry B.
This journal is © The Royal Society of Chemistry 2025

Supporting Information for

Structure-reactivity based control of radical-mediated degradation in thiol-Michael hydrogels

Bruce E. Kirkpatrick,[a,b,c] Miranda T. Rubio,[d] Naomi V. Elmer,[e] Tvishi Yendamuri,[f] Danielle S.W. Benoit,[g] C. Allan Guymon,[e] Kristi S. Anseth,[a,b] Tayler S. Hebner*[d]

*corresponding author, e-mail: thebner@purdue.edu

[a] Department of Chemical and Biological Engineering, University of Colorado Boulder, 596 UCB, Boulder, CO, 80309 (USA)

[b] BioFrontiers Institute, BioFrontiers Institute, University of Colorado Boulder, 596 UCB, Boulder, CO, 80309 (USA)

[c] Medical Scientist Training Program, University of Colorado Anschutz Medical Campus, 13001 East 17th Place, Aurora, Colorado, 80045 (USA)

[d] Davidson School of Chemical Engineering, Purdue University, 480 Stadium Mall Drive, West Lafayette, IN 47906

[e] Department of Chemical Engineering, Brigham Young University, Provo, UT 84604

[f] Department of Biochemistry, University of Colorado Boulder, 596 UCB, Boulder, CO, 80309 (USA)

[g] Department of Bioengineering, University of Oregon, 6231 University of Oregon, Eugene, OR 97403

Contents

Supplementary Figures S1-S13

Captions for Movies S1-2

Supplementary Figures

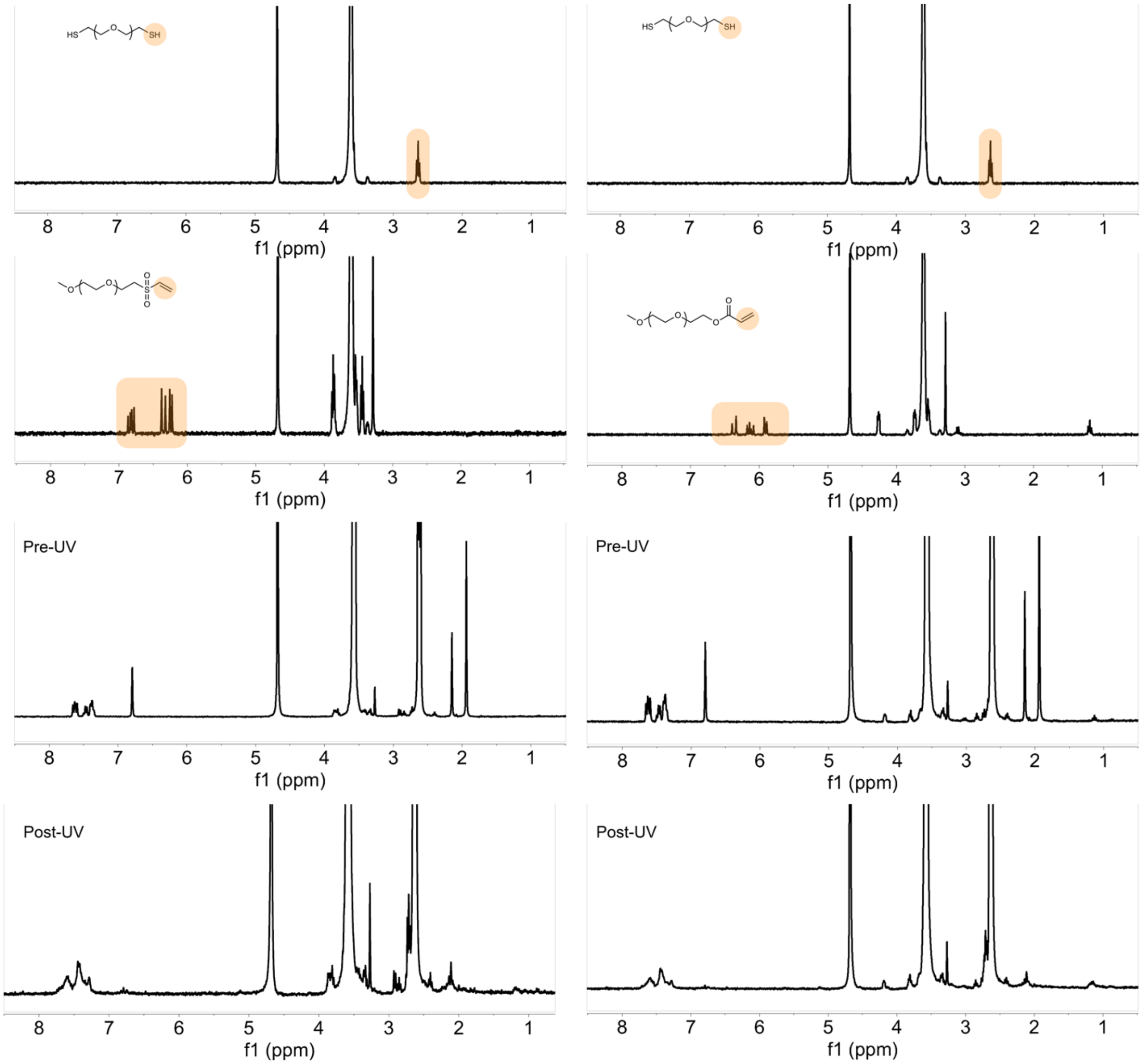

Fig. S1 | NMR of linear polymer alkene and thiol starting materials and thiol-Michael products before and after exposure. Disappearance of alkene protons (highlighted between 7.0-5.5 ppm) and thiol protons (highlighted at ~2.75 ppm) but retention of methyl group peak (~3.3 ppm) indicates essentially quantitative conversion of functional groups to thioethers. Post-UV (in the presence of LAP), spectra broaden and become noisy, making precise identification of molecular degradation products challenging.

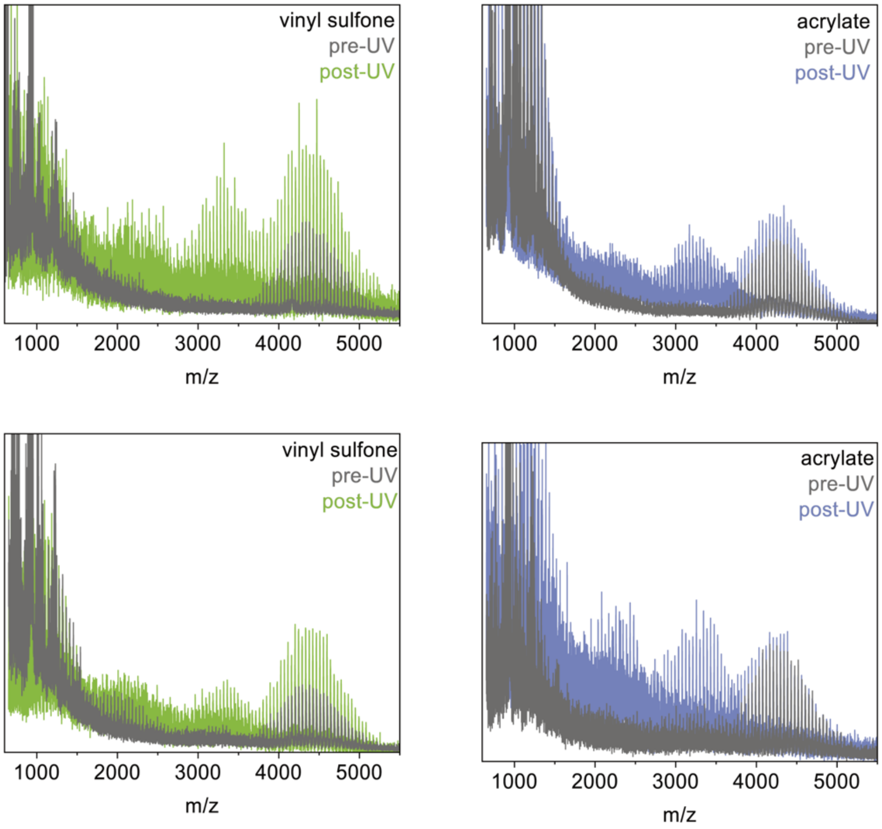

Fig. S2 | Complete MALDI spectra of samples prepared with 0.3 M (top) and 0.05 M (bottom) triethanolamine, with 4 kDa species before irradiation indicating the presence of Michael adduct species and lower-MW species emerging following light exposure in the presence of LAP.

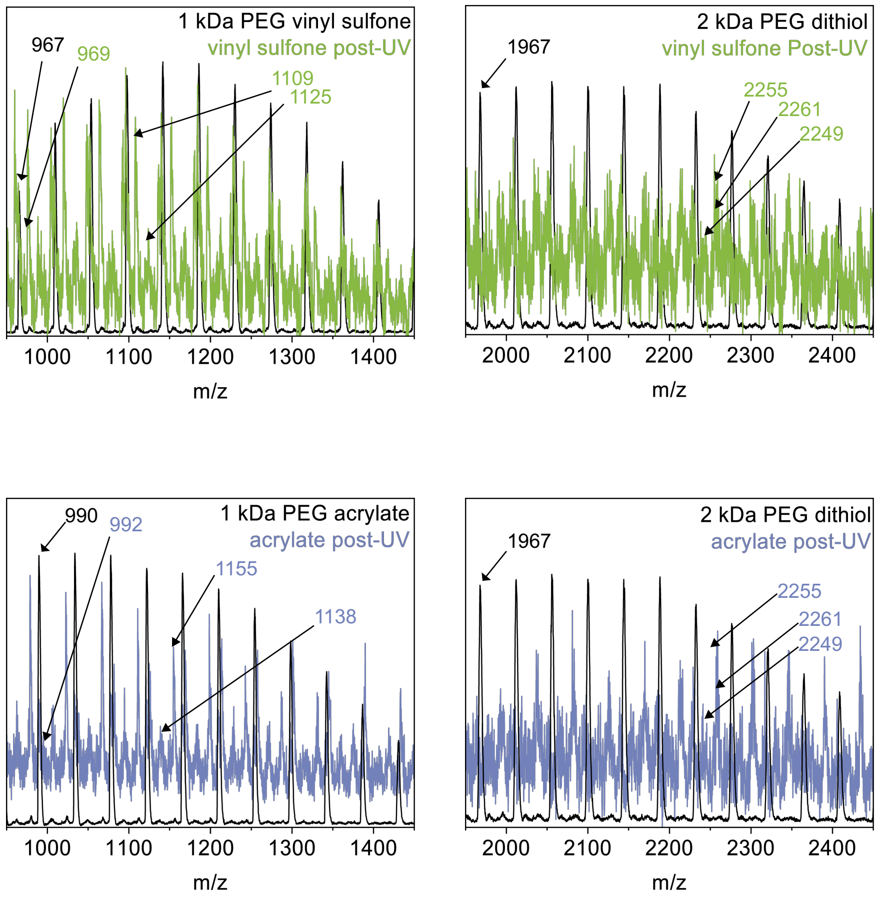

Fig. S3 | MALDI spectra of thiol-Michael adducts prepared with 0.05 M triethanolamine. Peaks indicating product complexes with triethanolamine are less pronounced than those in Fig. 2 (0.3 M triethanolamine) but identified product peaks are consistent.

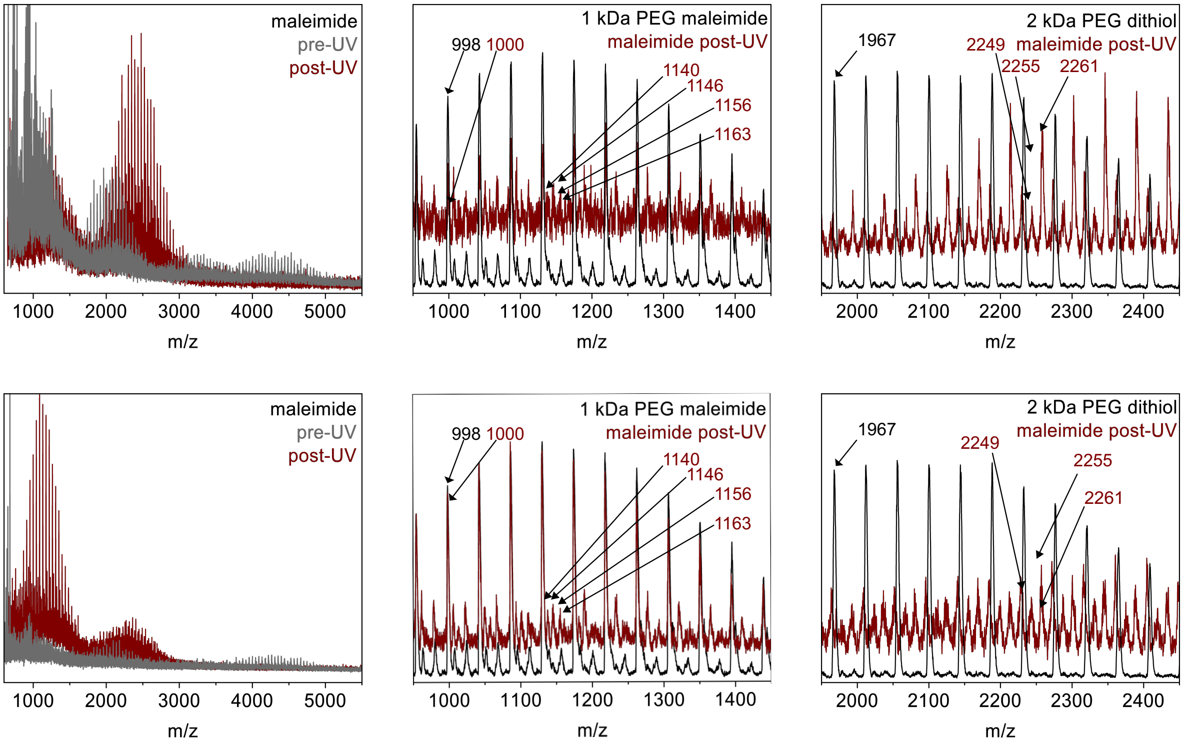

Fig. S4 | MALDI spectra of thiol-maleimide adduct degradation; polymers prepared with 0.3 M (top) and 0.05 (bottom) triethanolamine. Full spectra (left) reveal disappearance of 4 kDa product peak after irradiation in the presence of LAP, suggesting complete decomposition of thiol-Michael adducts. Magnified view of 1 kDa (alkene) starting materials and products (middle) shows alkyl products and complexes with initiator fragments and triethanolamine. Magnified view of 2 kDa (dithiol) starting materials and products (right) shows similar trends, with combinations of initiator fragments accounting for the observed shifts in molecular weight.

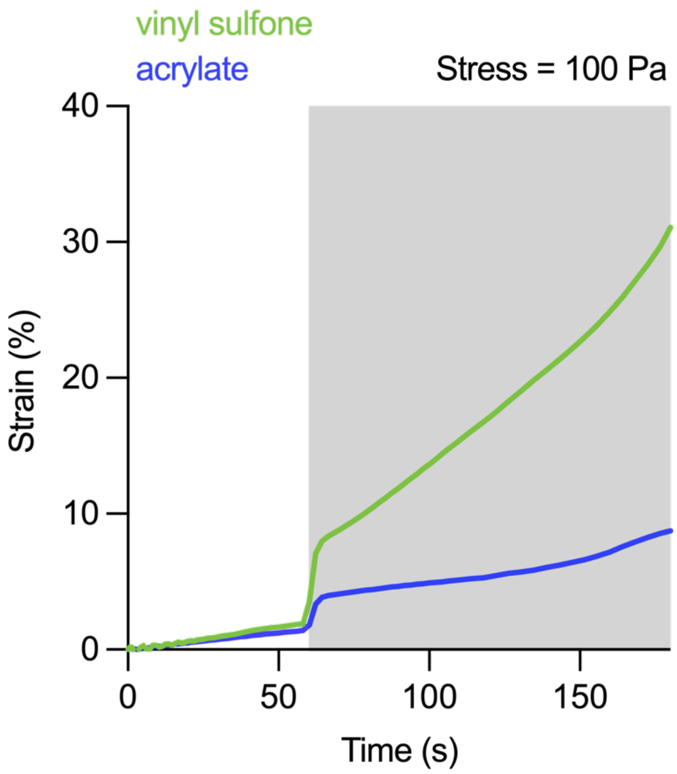

Fig. S5 | Photoinduced creep in on-stoichiometry thiol-Michael hydrogels formed from vinyl sulfone and acrylate precursors and containing 4 wt% LAP; vinyl sulfone shows dramatically increased creep, corresponding to enhanced softening responses seen with this chemistry. Shaded region = light on, 365 nm, 10 mW/cm^2. Strain data was baselined to 0 at t = 60 seconds in the raw data (shown above as t = 0) to eliminate noise from the creep measurement stabilizing.

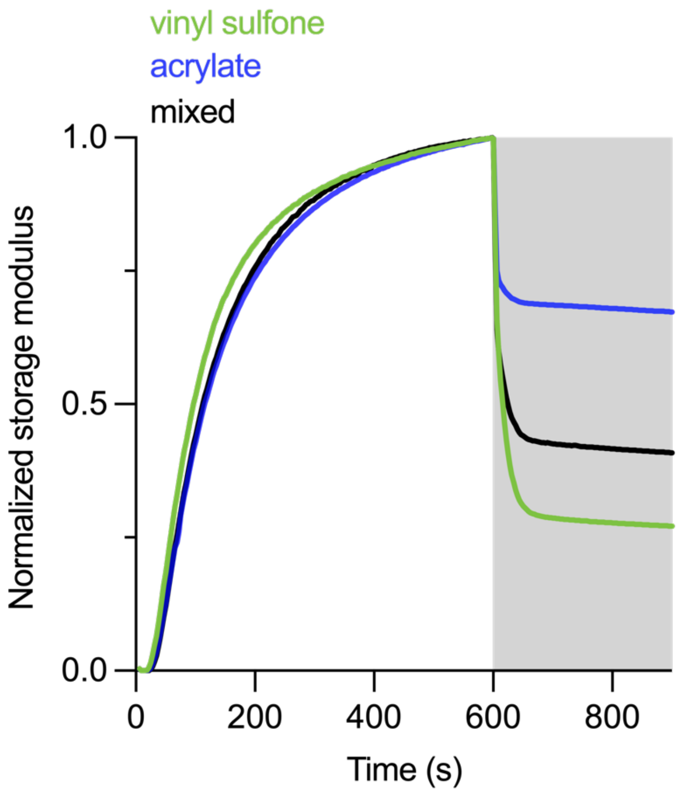

Fig. S6 | Photosoftening of on-stoichiometry thiol-Michael hydrogels containing isolated and mixed (1:1) alkene macromers and 2 wt% LAP; mixed macromers show an intermediated degradation profile. Shaded region = light on, 365 nm, 100 mW/cm^2.

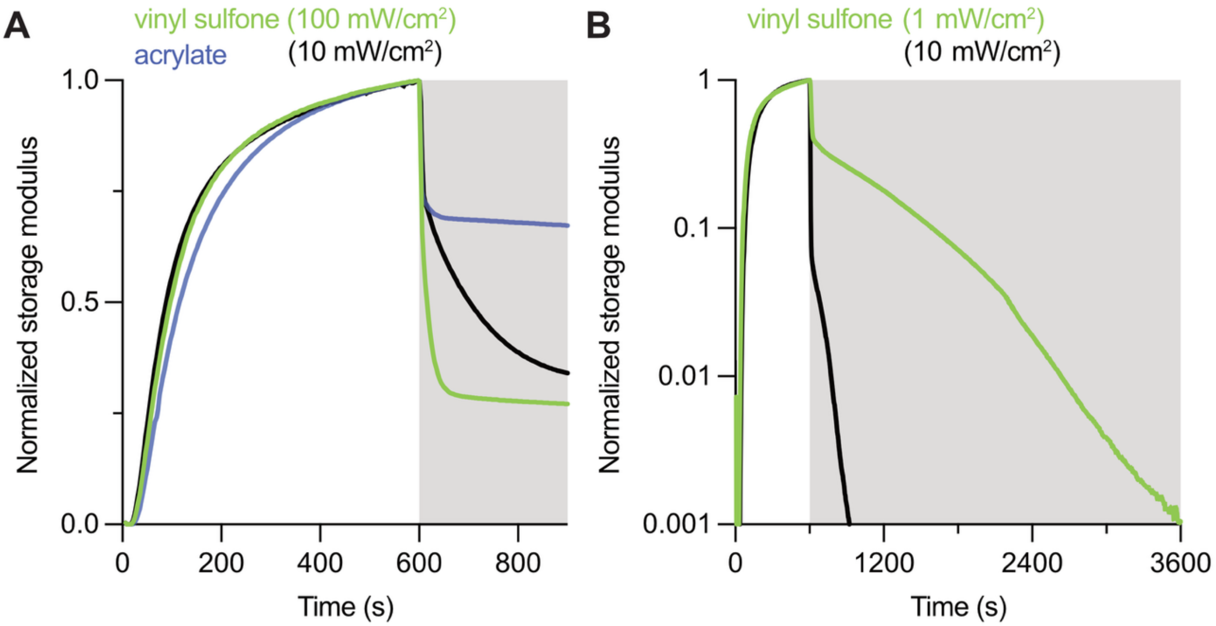

Fig. S7 | Photosoftening of thiol-Michael hydrogels with alternative light intensities. (A) High-intensity (100 mW/cm^2) irradiation does not increase the extent of degradation in hydrogels containing 2 wt% LAP. The y-axis is linearly scaled to more clearly illustrate relative differences in the same order of magnitude. (B) Low-intensity (1 mW/cm^2) irradiation does not decrease the extent of degradation in vinyl sulfone-crosslinked hydrogels containing 4 wt% LAP. The y-axis is log10 scaled to demonstrate the timescale across which order-of-magnitude changes occur with varied intensity. Shaded region = light on. Black lines in both panels correspond to the 10 mW/cm^2 control.

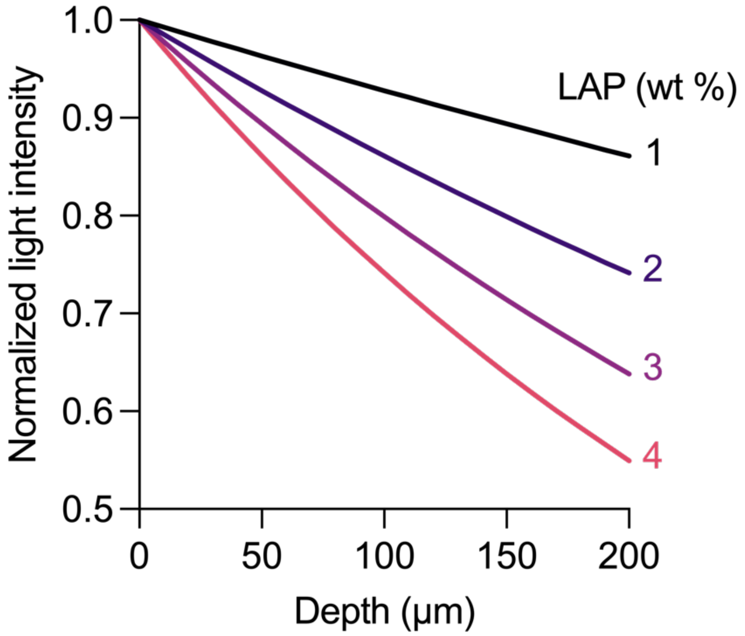

Fig. S8 | Estimated attenuation (according to Beer-Lambert law and a known LAP absorbance of 218 M^{-1} cm^{-1} at 365 nm) through 200 μm-thick hydrogels containing LAP (1-4 wt%). Optical thickness is typically defined as ≥20% attenuation, meaning that samples containing 2-4 wt% LAP are not optically thin.

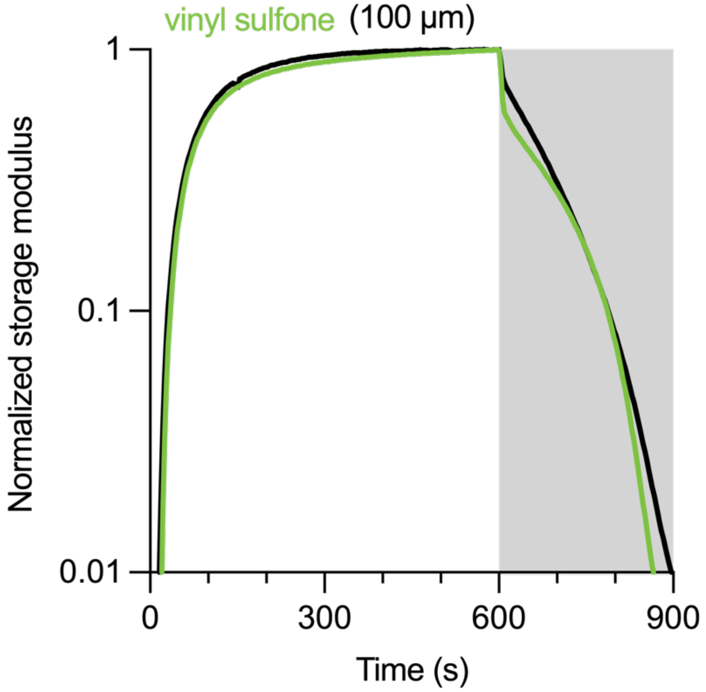

Fig. S9 | Photodegradation of thiol-vinyl sulfone hydrogels containing 4 wt% LAP at varied thickness. A 100 µm-thick hydrogel (black line) shows a very similar degradation profile to the 200 µm-thick reference gels used in this study (green line), suggesting that optical density does not preclude photodegradation (i.e., even ~40% attenuation provides sufficient light intensity to facilitate relatively rapid photodegradation). Shaded region = light on, 365 nm, 10 mW/cm^2.

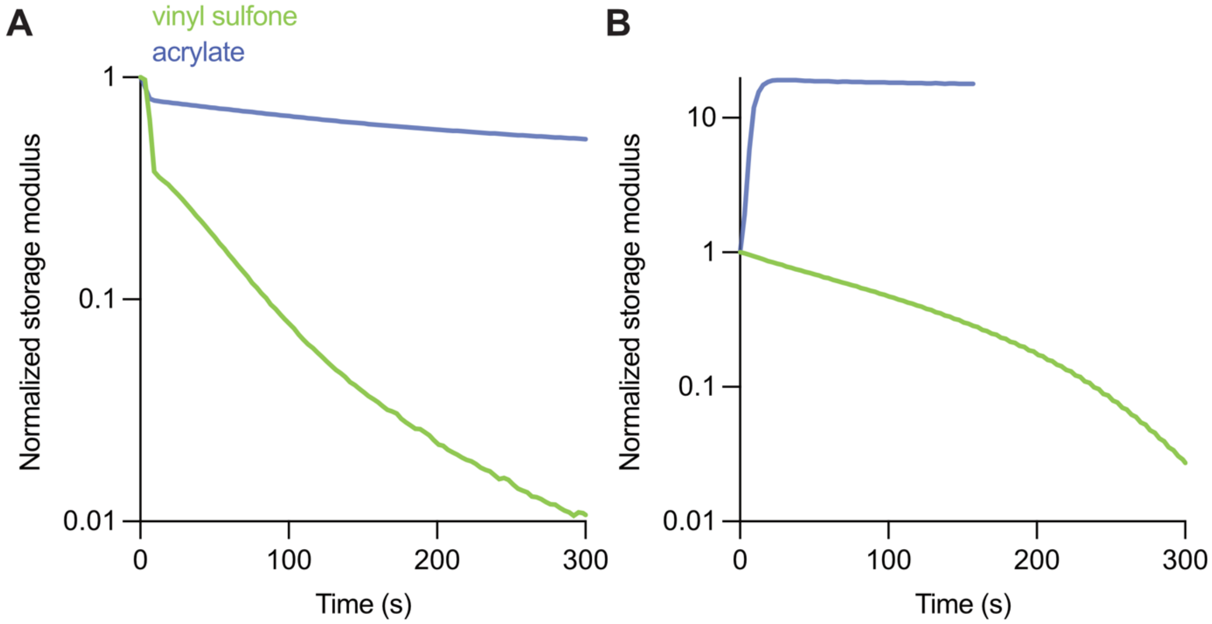

Fig. S10 | Responses to photoinitiated radicals in off-stoichiometry thiol-Michael hydrogels. (A) hydrogels containing excess thiol (2:1 thiol:alkene) and 4 wt% LAP show similar behavior to on-stoichiometry hydrogels, with increased degradation in vinyl sulfone versus acrylate gels, (B) hydrogels containing excess alkene (1:2 thiol:alkene) and 4 wt% LAP show differential responses to radicals, with softening in vinyl sulfone gels and stiffening in acrylate gels due to increased susceptibility to homopolymerization of the unreacted alkene. Light on at 0 seconds, 365 nm, 10 mW/cm^2.

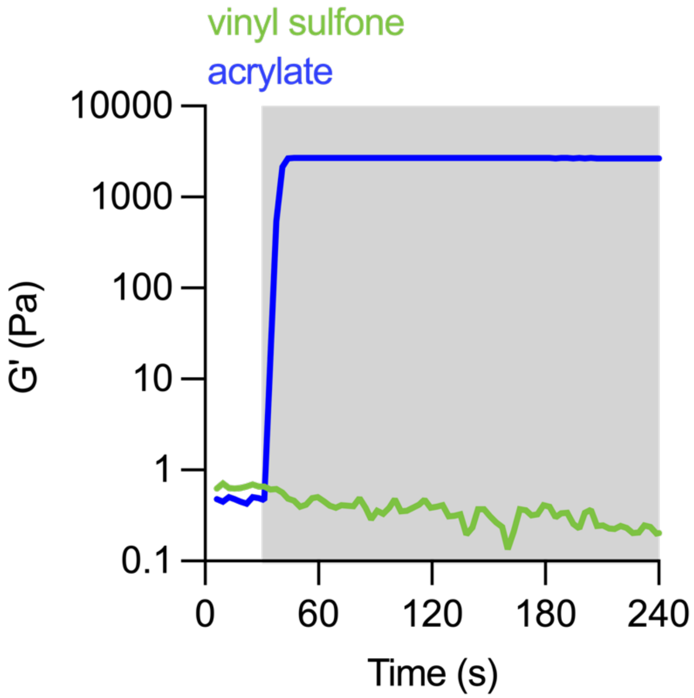

Fig. S11 | Homopolymerization of alkene macromers in the presence of 4 wt% LAP. Acrylates readily crosslink while vinyl sulfones show no gelation within 3.5 minutes of irradiation. Shaded region = light on, 365 nm, 10 mW/cm^2. Hydrogels were prepared in the same manner as thiol-Michael gels (10 mM alkene), excluding the thiol component.

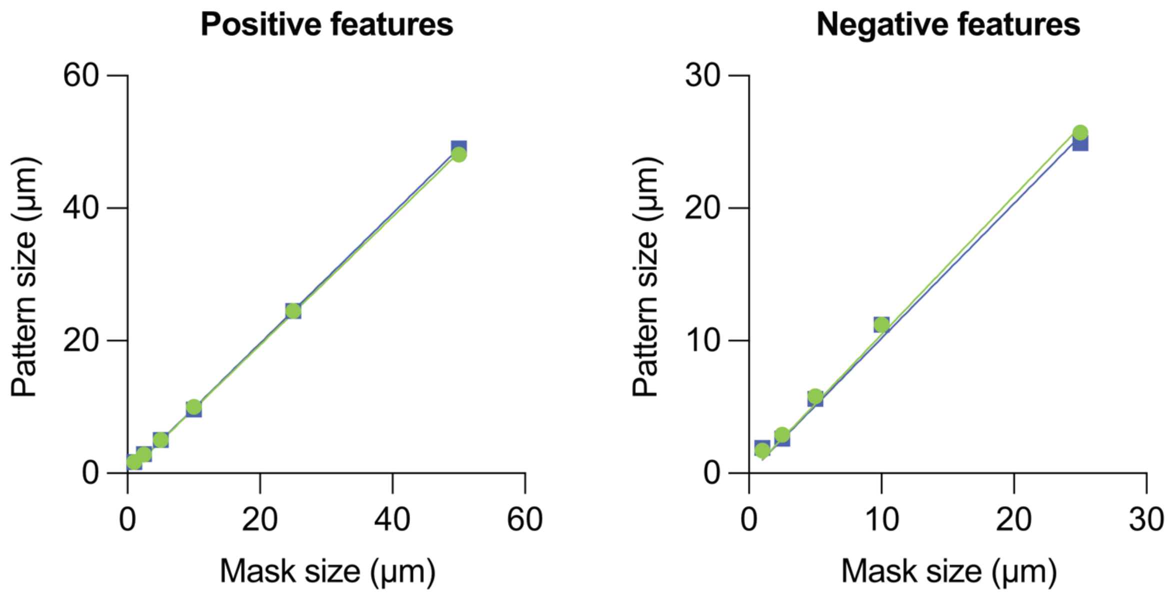

Fig. S12 | Quantified resolution of micron-scale photodegradation in vinyl sulfone (green) and acrylate (blue) thiol-Michael hydrogels. Both chemistries support high-fidelity patterning of positive and negative features, with $R^2 \geq 0.99$ for all linear fits.

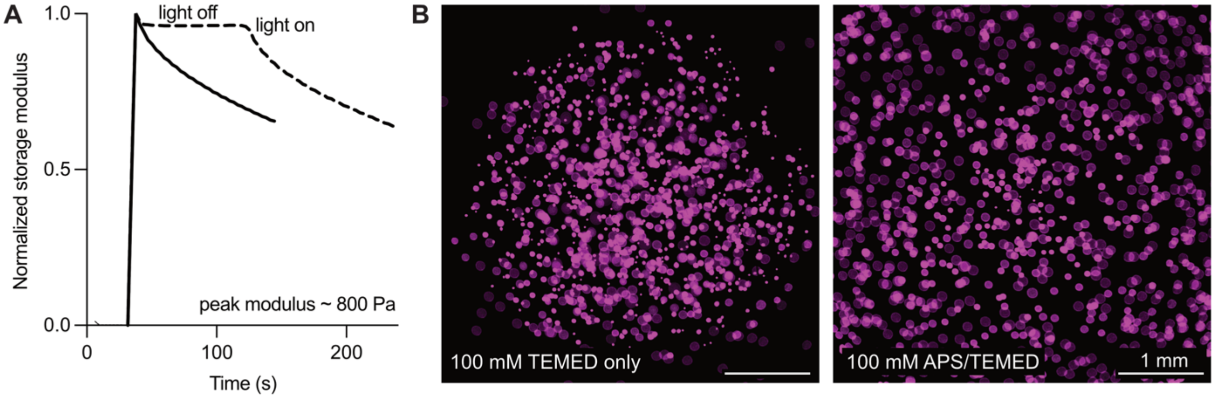

Fig. S13 | Radical-mediated thiol-ene polymerization and subsequent degradation of 4-arm 20 kDa PEG-norbornene and stoichiometric 2 kDa PEG-dithiol (6 wt% overall solids, 10 mM functional groups). (A) Photocrosslinking and photosoftening in the presence of 2 wt% LAP. Temporal control over softening response (dashed line) indicates that this behavior is not due to passive thermal relaxation. Light on at 30 seconds, 365 nm, 10 mW/cm2. (B) TEMED alone does not result in bead release from a thiol-ene hydrogel after 40 minutes of equilibration (left), while the addition of equimolar APS results in hydrogel degradation and fluorescent bead release (right).

Movie Captions

Movie S1 | APS/TEMED-based degradation of on-stoichiometry thiol-Michael hydrogels. 10 minute time-lapse of radical-mediated bead release from vinyl sulfone (left) and acrylate (right) hydrogels. Encapsulated beads are 1 μm FluoSpheres. Video dimensions are approximately 4.5 x 4.5 mm (i.e., gels are roughly 3 mm in diameter). On-stoichiometry formulations show similar responses, with both gels releasing beads upon addition of APS.

Movie S2 | APS/TEMED-based degradation of off-stoichiometry thiol-Michael hydrogels (1:2 thiol:alkene). 10 minute time-lapse of radical-mediated bead release from vinyl sulfone (left) and acrylate (right) hydrogels. Encapsulated beads are 1 μm FluoSpheres. Video dimensions are approximately 4.5 x 4.5 mm (i.e., gels are roughly 3 mm in diameter). Off-stoichiometry formulations show differential responses to radicals, with vinyl sulfones uniquely releasing beads upon addition of APS due to gel-stabilizing homopolymerization in the acrylate system.
